# Adverse Childhood Experiences and Cardiovascular Outcomes in Adult Congenital Heart Disease: ACEs in ACHD

**DOI:** 10.1016/j.jacadv.2025.101809

**Published:** 2025-05-15

**Authors:** David J. Harrison, Rania A. Mekary, Shilpa Vijayakumar, Erin Lake, Joseph Kay, Roni M. Jacobsen, Camila Londono-Obregon, Elizabeth Yeung, Sarah Kelly, Ann Poteet, Michael J. Landzberg, Molly Wallrich, Amber D. Khanna

**Affiliations:** a Boston Children's Hospital Cardiovascular Intensive Care Unit, Boston, Massachusetts, USA; b University of Colorado Adult and Teen Congenital Heart (C.A.T.C.H.) Program, Aurora, Colorado, USA; c Dartmouth-Hitchcock Adult Congenital Heart Disease Program, Lebanon, New Hampshire, USA; d Harvard T.H. Chan School of Public Health, Boston, Massachusetts, USA; e Massachusetts College of Pharmacy and Health Sciences, Boston, Massachusetts, USA; f Division of Cardiology, Brigham and Women's Hospital, Boston, Massachusetts, USA; g Boston Adult Congenital Heart (B.A.C.H.) Program, Boston Children's Hospital, Boston, Massachusetts, USA

**Keywords:** ACHD, adult congenital heart disease, adverse childhood experiences, psychosocial outcomes, quality of life, risk factors

## Abstract

**Background:**

Adverse childhood experiences (ACEs) are linked with poor physical and psychosocial health outcomes in adulthood, including cardiovascular disease.

**Objectives:**

The purpose of this study was to evaluate associations between ACEs and cardiovascular outcomes in adult congenital heart disease (ACHD).

**Methods:**

Outpatients with ACHD completed surveys including medical/psychosocial history, ACEs (range, 0-10), linear quality of life score (QoL, range, 0-100), and NYHA functional class (NYHA FC). Multivariable regression was performed on the exposure (ACEs score) on a binary composite outcome of self-reported heart failure, stroke, unplanned cardiac hospitalization, or emergency department visit for a cardiac cause. Secondary multivariable analyses included ACEs vs NYHA FC, and QoL score. Potential confounders included age, sex, ACHD complexity, number of prior surgeries, and mental health diagnosis.

**Results:**

A total of 153 respondents provided complete data. Seventy-eight percent had moderate or complex ACHD. Mean ACEs score was 2.26 ± 2.4, 41 (27%) reported ≥4 ACEs. Ninety-one (59%) met the composite outcome, of whom mean ACEs 2.68 ± 2.5. Each 1-U increase in ACEs was independently associated with 1.24 times odds of the composite outcome (95% CI: 1.04-1.49; *P* = 0.02), 1.19 times the cumulative odds of being in a worsened NYHA FC (95% CI: 1.03-1.37; *P* = 0.02), and 1.35 points lower QoL score (95% CI: −2.58 to −0.11; *P* = 0.03).

**Conclusions:**

In ACHD, ACEs appear common and were associated with higher odds of the composite outcome of heart failure, stroke, unplanned hospitalization, or emergency department visit due to heart condition, as well as worsened NYHA FC, and a lower quality of life score.

Congenital heart defects represent the most common clinically significant birth defect, often requiring surgical intervention in infancy or childhood. With advances in medical and surgical techniques, over 90% of infants born with congenital heart defects are expected to survive into adulthood.^1^ There are over 2.4 million long-term survivors living with adult congenital heart disease (ACHD) in the United States, and this population continues to grow.^2^^,^^3^ Many studies have shown a significant impact on the mental health of these individuals, often due to repeated hospitalizations, surgeries, isolation from their peers, and familial stressors. Coexisting mental health conditions are found in up to 50% to 65% of adults with congenital heart disease (CHD) and are associated with increased hospitalizations and lower quality of life.^4^, ^5^, ^6^, ^7^, ^8^, ^9^, ^10^ Beyond heart disease itself, studies have shown that noncardiac life events may be an additional source of lifelong stressors in patients and families with CHD.^11^, ^12^, ^13^

The adverse childhood experiences (ACEs) survey is a commonly used instrument to assess 10 psychosocial stressors that may have occurred in childhood and was designed to monitor how these stressors impact long-term health outcomes into adulthood.^14^ A higher ACEs score has been associated with a greater burden of cardiometabolic diseases and poor mental health; estimates vary by study methodology and cutoff scores, but examples include a 1.3 to 1.7 times increased risk of ischemic heart disease, 1.3 times the odds of diabetes, a 44% increase in depression, and a possible 1.7 to 2.4 times increased risk of early mortality.^15^, ^16^, ^17^, ^18^, ^19^, ^20^, ^21^ The American Heart Association (AHA) suggests interval screening for ACEs during clinical encounters, underscoring the strong connection between ACEs and cardiovascular outcomes.^22^ We hypothesize that the ACHD patient population may exhibit a high prevalence of ACEs and aim to test the association between a higher ACEs score and worsened cardiac outcomes specifically in ACHD.

## Methods

Patients ≥18 years of age with a diagnosis of CHD were recruited from outpatient encounters at one adult and one pediatric tertiary care center that provide specialized ACHD on the same medical campus. Recruitment materials were distributed with clinic intake paperwork over a 6-month period (February 1, 2022-July 1, 2022). Participants scanned a QR code with their smartphone, to direct them to the survey website, where informed consent was obtained prior to continuation with the survey. Data were collected via an encrypted, Health Insurance Portability and Accountability Act-compliant REDCap database hosted by the University of Colorado (version 13.1.18, Vanderbilt University).^23^ This study was reviewed and approved by the Colorado Multiple Institutional Review Board.

All responses, including those pertaining to medical history, were collected via self-report. The complete survey consisted of 3 sections. The first section included 40 items related to demographics, medical/surgical/psychiatric history, experience with mental health treatment, social and familial factors, and heart failure, stroke, unplanned hospitalizations, and emergency department (ED) visits specifically due to one's heart condition. CHD anatomic complexity was assigned to each respondent by a member of the study team based on the 2018 American College of Cardiology/AHA adults with CHD guidelines.^24^ Additional items included a 100-point linear quality of life (QoL) scale, three-item Oslo Social Support Scale, NYHA functional class (NYHA FC), medication nonadherence (defined as forgetting to take one's medications at least once per week), and lapses in care (defined as ever having a period of time >3 years without seeing a cardiologist).^25^^,^^26^ The second section was the post-traumatic stress disorder (PTSD) Checklist for the Diagnostic and Statistical Manual of Mental Disorders 5th-edition (PCL-5), with results reported separately.^27^ The third and final section was the ACEs survey, querying on exposures to any of 10 adverse experiences before age 18 in a binary yes/no fashion, resulting in a cumulative score of 0 to 10 (Central Illustration). Due to the sensitive content of these surveys, all survey items were optional, and patient identifiers were not collected to maximize patient privacy.

**Central Illustration:**
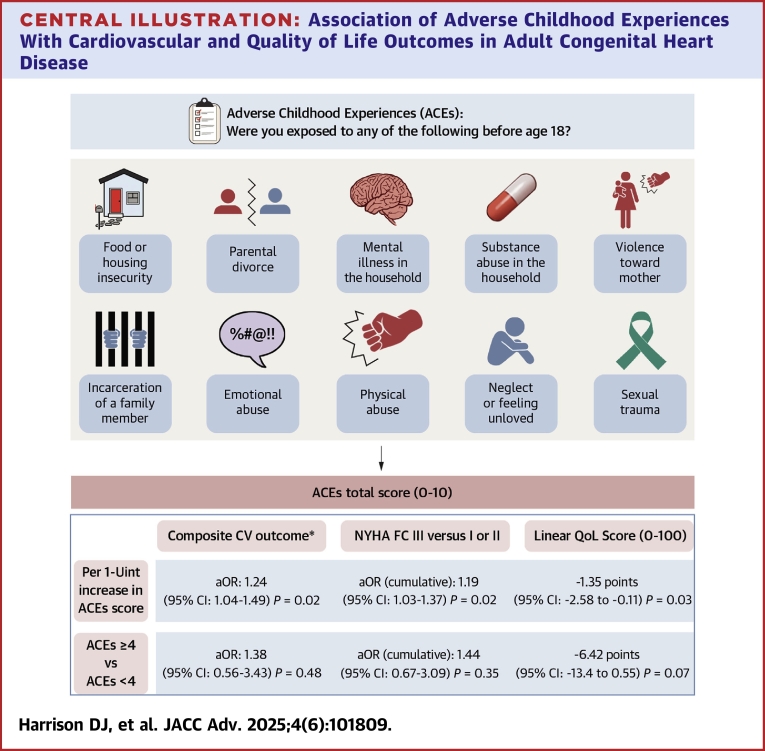
Association of Adverse Childhood Experiences With Cardiovascular and Quality of Life Outcomes in Adult Congenital Heart Disease Ten-item adverse childhood experiences (ACEs) survey results in adults with congenital heart disease, and the associated statistical estimate per one-unit increase in ACEs score or dichotomous ACEs ≥4 with a composite cardiovascular (CV) outcome (primary endpoint), a worsened NYHA FC, and a decrease in linear QoL score (secondary endpoints). ∗Composite CV outcome includes self-reported heart failure, stroke, unplanned hospitalization due to a cardiac cause, or emergency department visit due to a cardiac cause. aOR = adjusted OR; NYHA FC = NYHA functional class; QoL = quality of life.

### Statistical analysis

The exposure of interest was the number of ACEs, which was modeled separately as both a continuous variable (main analysis) and a dichotomous categorical variable (≥4 ACEs or <4 ACEs) (secondary analysis). The original ACEs validation studies suggested a binary cutoff of ≥4 ACEs as showing the greatest association with poor health outcomes,^14^ although more recent studies suggest reporting outcomes per 1-U increase in ACEs, thus both were assessed.^28^ The primary outcome of interest was a composite of heart failure, stroke, ED visits for a cardiac reason, or unplanned hospitalization. Each component of the composite outcome was dichotomous (yes/no) for a self-reported lifetime event, and the composite outcome itself was also a dichotomous endpoint for each patient reporting one or more components. Secondary outcomes included NYHA FC (I-IV) symptoms, self-reported by respondents using approved language from the AHA,^29^ and a 100-point linear QoL scale with a higher score indicating a better quality of life. Continuous variables were presented as mean ± SD, while categorical variables were presented as frequency with percentage. ACEs score was presented as mean ± SD as a descriptive value.

### Primary outcome

For the analysis of the composite adverse cardiovascular endpoints, multivariable logistic regression was used to model the association between ACEs in relation to the composite outcome, adjusting for the potential confounders of age and sex, complexity of CHD, number of prior surgeries, and coexisting mental health diagnosis (a binary composite of anxiety, depression, and PTSD). Several regression models were tested to evaluate for nonlinearity of data, including unadjusted, age-adjusted (linear), multivariable and age-adjusted (quadratic), and multivariable and age-adjusted (cubic spline, with one knot at median age). The ACEs exposure was additionally modeled as a linear vs quadratic term as a test of nonlinearity. The model with the lowest Akaike information criteria was selected as demonstrating the best fit.

### Secondary outcomes

For the secondary ordinal outcome of NYHA FC, a test of proportional odds was confirmed, and cumulative odds of reporting NYHA FC III vs II or I symptoms per 1-U increase in ACEs score was calculated. For the secondary continuous outcome of the QoL scale, linear model assumptions were confirmed, and a linear regression model was used to estimate the average change in QoL score per one-unit increase in ACEs. Secondary outcome measures of effect are reported with 95% CIs, adjusting for demographic and clinical confounders described above. Similar to the primary outcome, sequential tests of model complexity were performed for secondary outcomes and the model with the lowest Akaike information criteria was selected as best fit to the data. Cumulative OR was calculated via ordinal logistic regression for NYHA FC, and estimated change in QoL was calculated via linear regression.

## Results

A total of 220 patients accessed the survey, of which 14 opted out at consent. A total of 153 provided complete responses to the ACEs survey, resulting in a completion rate of 70%. Clinical and psychosocial characteristics of these 153 respondents are summarized in Table 1. The mean age was 35.4 ± 13.9 years. Forty-one respondents (27%) reported 4 or more ACEs, and the mean ACEs score among the entire cohort was 2.26 ± 2.42. One hundred and four out of 153 (68%) reported at least one ACE. A total of 91 patients (59%) reported at least one of the composite endpoints of stroke, heart failure, unplanned hospitalization, or ED visit, which are summarized in Table 2. Of the 91 who experienced the composite endpoint, 27 (30%) reported 4 or more ACEs, and the mean ACEs score was 2.68 ± 2.52. The most common reported ACEs included parental separation or divorce (N = 69), substance use in the household (N = 51), and mental illness in the household (N = 49).

**Table 1:** Baseline Characteristics

	Overall (N = 153, 100%)	ACEs <4 (n = 112, 73%)	ACEs ≥4 (n = 41, 27%)	ACEs Score
Age, y	35.4 ± 13.9	34.1 ± 12.9	38.8 ± 15.9	NA
Sex (at birth)				
Female	94 (61)	63 (56)	31 (76)	2.63 ± 2.52
Male	58 (38)	48 (43)	10 (24)	1.71 ± 2.14
Race				
Native American or Alaskan Native	2 (1)	2 (2)	0	a
Asian	3 (2)	3 (3)	0	a
Black or African American	4 (3)	1 (1)	3 (7)	a
White or Caucasian	126 (82)	90 (80)	36 (88)	2.35 ± 2.42
Other	9 (6)	9 (8)	0	a
Prefer not to state	4 (3)	3 (3)	1 (2)	a
Hispanic/Latinx	25 (16)	19 (17)	6 (15)	1.92 ± 2.40
Queer-identifying	18 (12)	10 (9)	8 (20)	3.44 ± 2.81
CHD complexity				
Simple	24 (16)	17 (15)	7 (17)	2.00 ± 2.57
Moderate	85 (56)	61 (54)	24 (59)	2.37 ± 2.31
Complex	34 (22)	27 (24)	7 (17)	2.29 ± 2.47
Unknown	10 (7)	7 (6)	3 (7)	1.90 ± 3.00
Lifetime number of interventions	3.5 ± 3.9	3.6 ± 4.2	3.1 ± 2.8	NA
NYHA FC				
I	74 (48)	58 (52)	16 (39)	1.73 ± 2.22)
II	65 (42)	44 (39)	21 (51)	2.68 ± 2.41
III	14 (9)	10 (9)	4 (10)	3.14 ± 2.98
IV	0	0	0	a
Pacemaker/ICD	29 (19)	19 (17)	10 (24)	2.69 ± 2.44
Arrhythmia	41 (27)	26 (23)	15 (37)	2.93 ± 2.63
Medication nonadherence	31 (20)	19 (17)	12 (29)	3.23 ± 2.53
Lapse in care >3 y	48 (31)	29 (26)	19 (46)	3.02 ± 2.37
Missed a cardiologist appointment	24 (16)	15 (13)	9 (22)	3.21 ± 2.48
Presence of a physical disability	37 (24)	24 (21)	13 (32)	2.95 ± 2.81
Linear QoL score	77.9 ± 18.1	79.8 ± 16.5	72.7 ± 21.2	NA
Educational attainment				
Did not finish high school	3 (2)	0	3 (7)	a
Finished high school or GED	29 (19)	24 (21)	5 (12)	2.10 ± 2.32
Some college or trade school	37 (24)	25 (22)	12 (29)	2.60 ± 2.86
Graduated college	54 (35)	39 (35)	15 (37)	2.17 ± 2.10
Master's degree or higher	29 (19)	23 (21)	6 (15)	1.79 ± 2.16
Period of unemployment, not by choice	57 (37)	43 (38)	14 (34)	2.23 ± 2.24
OSS-3 score	10.5 ± 2.3	11.0 ± 2.1	9.2 ± 2.5	NA
OSS-3 support level (N = 151)				
Poor	30 (20)	16 (14)	14 (35)	3.7 ± 2.64
Moderate	67 (44)	50 (45)	17 (43)	2.13 ± 2.43
Strong	54 (36)	45 (41)	9 (23)	1.56 ± 1.79
Burdensome parental attachment	70 (46)	44 (54)	26 (63)	3.13 ± 2.58
Any reported mental health condition	92 (60)	61 (54)	31 (76)	2.62 ± 2.43
Anxiety	78 (51)	52 (46)	26 (63)	2.64 ± 2.53
Depression	71 (46)	44 (39)	27 (66)	2.87 ± 2.48
PTSD	20 (13)	13 (12)	7 (17)	2.60 ± 2.23

Values are mean ± SD or n (%).

ACEs = adverse childhood experiences; CHD = congenital heart disease; GED = general education test diploma; ICD = Implantable cardioverter-defibrillator; NYHA FC = NYHA functional class; OSS-3 = Oslo Social Support Scale; PTSD = post-traumatic stress disorder; QoL = quality of life.

a Items where n < 10 observations are omitted.

**Table 2:** Summary of Event Rates in Composite Outcome^a^

	Overall (N = 153)	ACEs <4 (n = 112)	ACEs ≥4 (n = 41)	ACEs Score
Composite outcome	91 (59)	64 (57)	27 (66)	2.68 ± 2.52
Heart failure	22 (14)	15 (13)	7 (17)	2.82 ± 2.32
Stroke	10 (7)	7 (6)	3 (7)	3.30 ± 2.95
Unplanned hospitalization	51 (33)	38 (34)	13 (32)	2.28 ± 2.43
Emergency department visit due to heart condition	84 (55)	61 (54)	23 (56)	2.54 ± 2.43

Values are n (%) or mean ± SD.

Abbreviation as in Table 1.

a Total event counts will be greater than total N due to some respondents experiencing >1 composite outcome.

After performing sequential tests of regression model fit, final variables included age modeled as a cubic spline, sex at birth, CHD complexity, number of cardiac interventions, and a history of mental health diagnosis (Supplemental Table 1). A test of ACEs as a quadratic regression term yielded nonsignificant results, thus ACEs were modeled as a linear exposure.

On multivariable-adjusted regression analysis, there was on average 24% increased odds of the composite endpoint (adjusted OR: 1.24; 95% CI: 1.04-1.49; *P* = 0.02) per one-unit increase in ACEs score (Table 3). When ACEs score was dichotomized to (<4 or ≥4), the relationship with the composite endpoint yielded an adjusted OR of 1.38 (95% CI: 0.56-3.43) which did not achieve statistical significance (*P* = 0.48) (Table 4).

**Table 3:** Point Estimates for Primary and Secondary Outcomes in Relation to Adverse Childhood Experiences Score

Outcome	Point Estimates (95% CI) per 1-U Increase in ACE Score	*P* Value
Composite outcome^a^	MV OR^b^: 1.24 (1.04-1.49)	0.017
NYHA FC II or III vs I	MV-cumulative OR: 1.19 (1.03-1.37)	0.018
Change in linear QoL score	MV slope: −1.35 (−2.58 to −0.11)	0.034

MV = multivariable-adjusted; QoL = quality of life; other abbreviations as in Table 1.

a Composite outcome includes self-reported heart failure, stroke, ED visits due to heart condition, or unplanned hospitalization due to heart condition.

b MV: adjusted for age modeled as a cubic spline, sex at birth, CHD complexity, number of cardiac interventions, and a history of mental health diagnosis.

**Table 4:** Point Estimates for Primary and Secondary Outcomes in Relation to Adverse Childhood Experiences Score

	Point Estimates (95% CI) for ACE Score ≥ 4 vs ACE Score <4	*P* Value
Composite outcome^a^	MV OR^b^: 1.38 (0.56-3.43)	0.484
NYHA FC II or III vs I	MV-cumulative OR: 1.44 (0.67-3.09)	0.349
Change in linear QoL score	MV slope: −6.42 (−13.4 to 0.55)	0.074

MV = multivariable-adjusted; QoL = quality of life; other abbreviations as in Table 1.

a Composite outcome includes self-reported heart failure, stroke, ED visits due to heart condition, or unplanned hospitalization due to heart condition.

b MV: adjusted for age modeled as a cubic spline, sex at birth, CHD complexity, number of cardiac interventions, and a history of mental health diagnosis.

The secondary outcome of NYHA FC revealed on average a 14% increase in cumulative odds of reporting NYHA FC III symptoms compared to reporting no limitations or mild limitations (NYHA FC I and II), for every unit increase in ACE score, after adjusting for the same potential confounders discussed above (adjusted cumulative OR: 1.19; 95% CI: 1.03-1.37; *P* = 0.02) (Table 3). When ACE score was dichotomized (<4 or ≥4), the association with NYHA FC yielded an adjusted cumulative OR of 1.44 (95% CI: 0.67-3.09), which did not achieve statistical significance (*P* = 0.35) (Table 4).

Multiple linear regression on the secondary outcome of linear QoL revealed that for every unit increase in ACEs score, there was a significant decrease in QoL, after adjusting for potential confounders (slope: −1.35; 95% CI: −2.58 to −0.11; *P* = 0.03) (Table 3). When ACEs score was dichotomized (<4 or ≥4), the relationship with QoL was no longer statistically significant (slope: −6.42; 95% CI: −13.4 to 0.55; *P* = 0.07) (Table 4); the direction of the association remained consistently inverse.

## Discussion

This is the first study to consider the relationship between ACEs and cardiovascular events in ACHD, to our knowledge. We present increases in ACEs in single-unit form, in addition to the previously adopted ≥4 ACEs cutoff. Three statistical methods were applied based on outcome type, using flexible modeling for the variable age (cubic spline), which was selected as the model of best-fit.

Our findings result from an outpatient survey of adults with CHD at a single tertiary care center, showing a significant association between each single-point increase in ACEs score and the composite endpoint of stroke, heart failure, unplanned hospitalization, or heart-related ED visit. Each one-point increase in ACEs score was also associated with a lower linear QoL scale score, and a worsened NYHA FC. The ACEs survey as initially reported in 1998 used a cutoff of ACEs ≥4 as higher risk,^14^ although more recent studies recommend using the survey as a graded rather than binary scale for more informative results,^28^ thus we find value in reporting both. When scores were dichotomized as ≥4 vs <4, the association was no longer statistically significant, although this may be due to insufficient statistical power to detect a significant effect of a 4-point difference.

When comparing our findings to the existing literature, one study has assessed ACEs in ACHD, with the primary aim to test for associations between ACEs and attachment styles rather than cardiovascular outcomes.^30^ They showed in 100 ACHD patients, there was a mean ACEs score of 2.6, with 30% experiencing ≥4 ACEs.^30^ This is overall similar but slightly higher than our data showing a mean of 2.3, and 27% reporting ≥4 ACEs. Collectively, the prevalence of ACEs in ACHD seems higher than the general U.S. population, as studied by the Centers for Disease Control and Prevention, showing a mean ACEs score of 1.56, and 17.3% reporting ≥4 ACEs, in over 260,000 respondents.^31^^,^^32^

Because data on ACEs in ACHD are limited, it is difficult to draw conclusions on the underlying cause of the higher prevalence, except to acknowledge that many ACHD patients report substantial life stressors from noncardiac causes in addition to ACHD itself.^27^ Some potential stressors observed among children with CHD and their families include financial burdens, parental anxiety and PTSD, inconsistent caregivers, bullying, and social stigma.^11^^,^^33^^,^^34^ Fortunately, structured family support programs within pediatric heart centers are becoming more common,^35^ and our findings further underscore their critical importance.

Future studies on resilience in ACHD will be a highly important balancing measure to our findings on ACEs and stressors. ACEs do not need to be viewed as only a negative event. Resilience is defined as one's ability to withstand, recover, and adapt to stressors or trauma, and adults with CHD exhibit this quality in abundance. Higher resilience in ACHD has been associated with a slightly higher health-related quality of life score,^36^ while lower resilience is associated with higher symptoms of post-traumatic stress.^37^ Research is ongoing to test the efficacy of resilience-building interventions in the ACHD population,^38^ which may be especially valuable in a patient with a history of ACEs exposures.

As the ACHD population grows in number with extended life expectancy, we can anticipate an increasing trend in acquired heart disease leading to significant morbidity and mortality.^39^^,^^40^ Due to the strong associations between ACEs and acquired cardiometabolic conditions,^20^^,^^41^ alongside complications from lifelong CHD, individuals with ACHD and a history of ACEs may represent a vulnerable population in terms of poor outcomes, worsened quality of life, and increased health care needs. The AHA supports screening all patients for ACEs,^22^ although this can be performed in schools, primary care settings, or by clinical support staff, depending on resources. The cardiologist plays an important role through understanding how ACEs may impact the illness experience of ACHD patients, so we can better counsel about lifestyle recommendations, and refer for psychological support as indicated.

### Study Limitations

The cross-sectional study design limits the ability to establish causality between ACEs and cardiovascular outcomes, although the nature of ACEs having occurred in childhood does suggest a sequential relationship. Also, while this sample represents the general racial and ethnic demographics of the surrounding area, we lack the sample size to apply statistical analyses to racial and ethnic minorities who have historically experienced disproportionately high rates of both ACEs and poor cardiovascular outcomes.^32^^,^^42^ Although the choice to use a composite outcome as our primary endpoint increased statistical power, it is important to note that not all components of the composite outcome are of equal clinical significance. For example, the high prevalence of ED visits reflects a patient's self-reported perception of a cardiac cause over the entire lifespan, which likely includes some low-acuity and/or noncardiac encounters. However, we chose to include this outcome component as an important measure of health care utilization and patient illness experience,^11^ but acknowledge that it reduces the clinical severity of our composite outcome and may introduce some misclassification bias. Similarly, some authors have criticized the ACEs survey due to equal weighting of each item (1 point each), despite a potentially wide range in severity of any individual event.^28^ Finally, all survey items being optional resulted in some missing data (<10% for any covariate), and our use of self-report surveys are prone to self-selection bias, recall bias, and misclassification. Self-report of medical diagnoses would optimally be validated against the medical record in future studies, if patients are comfortable providing identifiable data.

## Conclusions

We have used 3 different regression strategies to show that in the ACHD population, ACEs appear to be common, and a higher ACEs score was associated with increased odds of the composite outcome of heart failure, stroke, unplanned hospitalization, or ED visit due to their heart condition, as well as a lower linear QoL score, and worsened NYHA FC. Future longitudinal studies, larger sample sizes, and testing of a comparison group possibly with other chronic conditions, will be highly valuable to establish a potential causal relationship. Overall, ACHD clinicians should be familiar with ACEs and how they may impact the cardiovascular health of their patients.

Perspectives **COMPETENCY IN MEDICAL KNOWLEDGE:** ACEs are associated with worse cardiovascular and quality of life outcomes in adults with CHD.

**TRANSLATIONAL OUTLOOK:** Awareness and assessment for exposure to ACEs may help identify ACHD patients who will benefit from additional specialized counseling services.

## Funding support and author disclosures

This work was supported by the 10.13039/100000002NIH/10.13039/100006108National Center for Advancing Translational Science, 10.13039/100011866Colorado Clinical and Translational Sciences Institute Grant Number UL1 TR002535 and Harvard T.H. Chan School of Public Health Professional Development Support Fund. The opinions expressed in this article are the authors' own and do not reflect the view of the National Institutes of Health, the Department of Health and Human Services, or the United States government. The authors have reported that they have no relationships relevant to the contents of this paper to disclose.
