# Supplementary material for: Adverse Childhood Experiences and Cardiovascular Outcomes in Adult Congenital Heart Disease: ACEs in ACHD
Source: JACC Adv. 2025 May 15;4(6):101809. doi: 10.1016/j.jacadv.2025.101809 (PMC12145704; doi:10.1016/j.jacadv.2025.101809)
Supplement: Supplementary data [file mmc1.docx]

| **Supplemental Table 1A**: Odds Ratios^1^ of composite outcome of heart failure, stroke, ED visits, or unplanned hospitalization. The model of best fit as evidenced by lowest AIC is italicized. | | | |
| --- | --- | --- | --- |
|  | **OR (± 95% CI) for a 1-unit increase in ACE score (continuous)** | **P-value** | **AIC** |
| Composite outcome, unadjusted | 1.22 (1.05, 1.42) | 0.011 | 203.4 |
| Composite outcome, age-adjusted | 1.21 (1.04, 1.41) | 0.013 | 205.3 |
| Composite outcome, MV-adjusted, with age, linear^2^ | 1.21(1.03, 1.42) | 0.019 | 195.5 |
| Composite outcome, MV-adjusted, with age, quadratic^3^ | 1.22 (1.04, 1.43) | 0.016 | 197.0 |
| *Composite outcome, MV-adjusted, with cubic spline on age with one knot at median age^4^* | *1.24 (1.04, 1.49)* | ***0.017*** | ***173.2*** |
|  | **OR (± 95% CI)* for ACE score ≥4** | **P-value** | **AIC** |
| Composite outcome, unadjusted | 1.45 (0.69, 3.05) | 0.332 | 209.6 |
| Composite outcome, age-adjusted | 1.41 (0.66, 3.00) | 0.371 | 211.4 |
| Composite outcome, MV-adjusted, with age, linear^2^ | 1.40 (0.62, 3.13) | 0.415 | 200.8 |
| *Composite outcome, MV-adjusted, with cubic spline on age with one knot at median age^4^* | 1.38 (0.56, 3.43) | 0.484 | 179.0 |
| **Supplemental Table 1B**: Cumulative odds ratios^1^ of moderate limitations in activity (NYHA Functional Class III) compared to the cumulative odds of having no or mild limitations (NYHA Functional Class I and II). | | | |
|  | **Cumulative OR (± 95% CI) for a 1-unit increase in ACE score (continuous)** | **P-value** | **AIC** |
| NYHA FC III versus I or II, unadjusted | 1.20 (1.05, 1.37) | 0.006 | 284.1 |
| NYHA FC III versus I or II, age-adjusted | 1.19 (1.04, 1.35) | 0.011 | 280.6 |
| NYHA FC III versus I or II, MV-adjusted, with age, linear^2^ | 1.16 (1.01, 1.33) | 0.033 | 270.1 |
| NYHA FC III versus I or II, MV-adjusted, with age, quadratic^3^ | 1.16 (1.01, 1.33) | 0.037 | 272.0 |
| *NYHA FC III versus I or II, MV-adjusted, with cubic spline on age with one knot at median age^4^* | *1.19 (1.03, 1.37)* | *0.018* | ***266.6*** |
|  | **Cumulative OR (± 95% CI)* for ACE score ≥4** | **P-value** | **AIC** |
| NYHA FC III versus I or II, unadjusted | 1.55 (0.78, 3.08) | 0.213 | 290.2 |
| NYHA FC III versus I or II, age-adjusted | 1.40 (0.70, 2.83) | 0.342 | 286.3 |
| NYHA FC III versus I or II, MV-adjusted, with age, linear^2^ | 1.26 (0.60, 2.63) | 0.543 | 274.1 |
| *NYHA FC III versus I or II, MV-adjusted, with cubic spline on age with one knot at median age^4^* | 1.44 (0.67, 3.09) | 0.349 | 271.3 |
| **Supplemental Table 1C**: Change in linear QoL score^1^ based on changes in ACEs. | | | |
|  | **Change in QoL score (± 95% CI) for a 1-unit increase in ACE score (continuous)** | **P-value** | **AIC** |
| Linear QoL score, unadjusted | -1.49 (-2.66, -0.32) | 0.014 | 1275.3 |
| Linear QoL score, age-adjusted | -1.40 (-2.57, -0.23) | 0.021 | 1274.9 |
| Linear QoL score, MV-adjusted, with age, linear^2^ | -1.09 (-2.24, 0.06) | 0.064 | 1265.4 |
| Linear QoL score, MV-adjusted, with age, quadratic^3^ | -1.00 (-2.16, 0.15) | 0.090 | 1265.3 |
| *Linear QoL score, MV-adjusted, with cubic spline on age with one knot at median age^4^* | *-1.35 (-2.58, -0.11)* | ***0.034*** | ***1113.6*** |
|  | **Change in QoL score (± 95% CI) for ACE score ≥ 4** | **P-value** | **AIC** |
| Linear QoL score, unadjusted | -7.03 (-13.50, -0.55) | 0.035 | 1277.0 |
| Linear QoL score, age-adjusted | -6.37 (-12.9, 0.13) | 0.057 | 1276.7 |
| Linear QoL score, MV-adjusted, with age, linear^2^ | -5.23 (-11.6, 1.13) | 0.110 | 1266.3 |
| *Linear QoL score, MV-adjusted, with cubic spline on age with one knot at median age^4^* | -6.42 (-13.4, 0.55) | 0.074 | 1115.0 |
| Note: ACEs as an exposure was also modeled as a quadratic term as a test of non-linearity of data, with nonsignificant results (P = 0.37 for the quadratic term), thus suggesting no evidence that the data would be better fit with nonlinear modeling.  ^1^Odds ratios (ORs) were derived from a multivariable logistic regression (1a), cumulative odds ratios (ORs) were derived from a multivariable ordinal logistic regression (1b), and changes in QoL score were derived from a multivariable linear regression. Results on the natural log scale of QoL score were similar to those on the raw QoL score, which are shown in the table (1c).  ^2^Adjusted for age (continuous), sex (male, female), CHD complexity (simple, moderate, complex, unknown), number of interventions (continuous), and coexisting mental health diagnosis (yes, no).  ^3,4^Adjusted for the same covariates in model 2 above in addition to flexible modeling for age (continuous), as either age-squared (3), or cubic spline (4).  ACE: Adverse Childhood Experiences, AIC: Akaike information criterion, MV: multivariable, NYHA FC: New York Heart Association Functional Class, QoL: Quality of Life | | | |
